# Optimizing Outcomes in Mangled Lower Extremity Reconstruction: Insights from a Retrospective Study of 93 Patients and Their Functional Scores

**DOI:** 10.3390/jcm14051436

**Published:** 2025-02-21

**Authors:** Serdar Düzgün, Mehmet Taner Özdemir, Nurettin Manti, Nuri Koray Ülgen, Mehmet Orçun Akkurt

**Affiliations:** 1 Department of Plastic and Reconstructive Surgery, Anadolu Medical Center Hospital, 41400 Kocaeli, Türkiye; serdarduzgun@gmail.com; 2 Department of Orthopedics and Traumatology, Anadolu Medical Center Hospital, 41400 Kocaeli, Türkiye; taner.ozdemir@anadolusaglik.org; 3 Department of Orthopedics and Traumatology, Ankara Bilkent City Hospital, 06800 Ankara, Türkiye; 4 Department of Orthopedics and Traumatology, Ankara Sincan Training and Research Hospital, 06949 Ankara, Türkiye; nurikoray@gmail.com

**Keywords:** lower extremity injury, reconstruction, MESS, Gustilo–Anderson, LEFS

## Abstract

**Background/Objectives:** Over the past 25 years, reconstructive techniques and patient management advancements have significantly improved outcomes in mangled lower extremity injuries. Functional results of limb salvage have been demonstrated to surpass those of primary amputations. Developments such as local fasciocutaneous flaps, vacuum-assisted closure, and hyperbaric oxygen therapy have enhanced the reconstructive ladder. Despite progress, the utility of the Mangled Extremity Severity Score (MESS) and Gustilo–Anderson classification remains debated, particularly in their prognostic value for limb salvage decisions. In the study, we aimed to evaluate the outcomes of optimizing mangled lower extremity reconstruction in 93 patients, focusing on their functional scores retrospectively. **Methods:** This retrospective study analyzed 93 patients treated for mangled lower extremities between January 2015 and October 2022. Patients were assessed for age, gender, injury location, MESSs, Gustilo–Anderson classifications, surgical methods, and functional outcomes using the Lower Extremity Functional Scale (LEFS). Surgical interventions included internal and external fixation, skin grafts, local flaps, muscle flaps, and free tissue transfer. LEFS scores were categorized into disability levels for functional evaluation. Correlations were drawn between LEFS and variables such as MESS, Gustilo–Anderson types, and nerve injuries. **Results:** Among the 93 patients, 16 had MESSs ≥ 7, and 77 had MESSs < 7. Reconstruction methods included local fasciocutaneous and muscle flaps (37 patients), free tissue transfer (29 patients), and skin grafting with vacuum-assisted closure (27 patients). Smoking was associated with delayed union and increased infection rates. LEFS scores were significantly lower in patients with MESSs ≥ 7, Gustilo grade 3C fractures, and tibial nerve injuries. Flap failures and a higher number of surgeries (>3) also correlated with poorer functional outcomes. The average soft tissue healing time was 18 days, and bone union time was 17 weeks. **Conclusions:** Lower extremity reconstruction demands precise surgical planning and execution, prioritizing functional restoration. MESSs and Gustilo–Anderson classifications provide practical frameworks but have limitations in predicting long-term functionality. Factors such as joint involvement, nerve injuries, and flap selection significantly influence outcomes. Smoking and delayed healing remain critical challenges. While free flaps are essential for complex defects, more straightforward methods yield better outcomes in suitable cases. LEFS emerged as a reliable tool.

## 1. Introduction

Over the past quarter-century, significant advancements have occurred in the reconstruction of severely injured lower extremities, the evaluation of patients, and treatment techniques. The most longstanding and crucial question in this field has been resolved, revealing that the functional outcomes of limb preservation are generally superior to immediate amputations in numerous respects [1]. Initially, traumatic soft tissue defects were thought to rely heavily on free flaps for treatment. The development of new axial patterns for flap techniques eventually led to successful outcomes with the local fasciocutaneous flaps [2,3,4]. Additionally, relatively recent methods like vacuum-assisted closure and hyperbaric oxygen therapy have emerged as beneficial aids in managing dressing changes, thus supporting the reconstructive process for such injuries [5,6].

The Mangled Extremity Severity Score (MESS) was initially developed to assess mangled lower extremities and predict the likelihood of amputation. Today, MESS is also applied to upper extremity injuries, demonstrating good sensitivity and specificity. When dealing with extremity trauma, using MESS is essential for evaluating the potential for limb salvage. MESS offers a dependable framework for making important decisions in surgical applications and treatments [7].

The Lower Extremity Functional Scale (LEFS) is a patient-reported tool used to assess functional status in individuals with lower extremity musculoskeletal issues. LEFS consists of 20 items, each scored from 0 to 4, where 0 indicates extreme difficulty or inability to perform the activity, and 4 indicates no difficulty at all. To obtain the total score, one simply sums the scores of the individual items. The maximum score of 80 means that there are no functional limitations, while a minimum score of 0 indicates significant limitations in function [7].

Additionally, the Gustilo–Anderson classification for open fractures enhances our ability to assess prognosis and develop effective treatment algorithms that address bone and soft tissue injuries while preventing infections. By carefully tailoring surgical interventions based on this vital information, we can optimize antibiotic use and select the most effective bone fixation and soft tissue reconstruction methods, ultimately improving patient outcomes.

Despite advances in medical technology, managing a severely injured limb is a complex decision-making process for the patient, their family, and the surgical team. Often, these injuries result from high-energy trauma that can also severely affect other organ systems, such as the brain, chest, and pelvis. Therefore, resuscitation and the management of any life-threatening injuries take priority over treating limb injuries (the principle of “life before limb”). As a result, definitive treatment for a mangled extremity, aside from primary amputation, is rarely indicated during the acute phase of care.

The objective of this study is to systematically analyze the factors influencing functional outcomes in patients undergoing reconstruction for mangled lower extremities. The research question guiding this investigation is as follows: “What are the key determinants of functional recovery in patients with mangled lower extremity injuries?” The hypothesis is that higher MESSs, Gustilo grade 3C fractures, and tibial nerve injuries are associated with poorer functional outcomes while smoking and the number of surgical interventions further exacerbate these effects.

This study found that patients with higher MESSs, Gustilo grade 3C fractures, and tibial nerve injuries exhibited significantly lower functional scores. Smoking was associated with delayed union and increased infection rates, complicating recovery. Additionally, flap failures and a higher number of surgeries were correlated with poorer functional outcomes. The average soft tissue healing time was 18 days, while bone union time averaged 17 weeks. This research provides valuable insights into the factors influencing functional recovery in mangled lower extremity reconstruction, offering a foundation for developing targeted interventions to optimize patient outcomes. This study evaluated the results using the Lower Extremity Functional Scale (LEFS). It was also aimed to determine the relationship between LEFS and factors such as MESS (Mangled Extremity Severity Score), the Gustilo–Anderson classification, and the anatomical specifics of injuries in a cohort of 93 patients.

## 2. Materials and Methods

This study was conducted in an area which is characterized by significant industrial activity and heavy traffic flow. Between January 2015 and October 2022, 115 patients were admitted to our clinic, undergoing salvage operations, lower extremity reconstruction, or amputation, determined by their initial Mangled Extremity Severity Score (MESS) at the time of admission. The primary causes of injury included motor vehicle accidents and workplace incidents. The inclusion criteria were established as follows: 93 patients were categorized in the salvage group, while 22 required amputations due to severe neurovascular damage, catastrophic soft tissue injuries, and additional life-threatening trauma. Notably, ten cases involved primary amputations, whereas twelve were classified as secondary amputations. Data collection was conducted meticulously through a retrospective review of electronic medical records.

Comprehensive demographic information, including age and gender, as well as details regarding the injuries such as location and mechanism were systematically compiled. Clinical assessments included MESSs and Gustilo–Anderson classifications. Surgical interventions were thoroughly documented, specifying the types and numbers of procedures performed. Functional outcomes were assessed using the Lower Extremity Functional Scale (LEFS), a validated instrument for evaluating physical function in patients with lower extremity musculoskeletal conditions; LEFS scores range from 0 to 80, with higher scores indicating better functional abilities.

The exclusion criteria for all groups included patients with incomplete medical records, those who did not undergo surgical interventions, and individuals with pre-existing conditions that could affect the assessment of functional outcomes, such as neurological disorders or systemic diseases impacting mobility. Consistent environmental factors were maintained throughout the study, including the hospital setting, the experience of the surgical team, and postoperative care protocols. The follow-up duration varied among patients, with a minimum requirement of twelve months post-injury to ensure a comprehensive assessment of functional recovery. Regarding age, the distribution ranged from 5 to 78 years, with an average of 34 years. Patients were categorized into two groups: those over 50 and those under 50. Additionally, seven patients were younger than 18 years.

Location of injury: The injury site was the leg and ankle region; therefore, patients suffered tibia/fibula and ankle region fractures.

Mangled Extremity Severity Score: MESS was evaluated at initial admission [8]. Patients were divided into two main groups, one with scores of 7 or above (≥7) and the other with scores of less than 7 (<7). 

Gustilo–Anderson classification: All patients had open fractures, and the Gustilo–Anderson classification was used for the treatment algorithm [9,10].

### 2.1. Surgical Interventions

Surgical interventions were categorized into five primary types: internal fixation, external fixation, skin grafting, local flap coverage, and free tissue transfer.

Internal Fixation: This intervention was utilized for the stabilization of fractures, involving the use of plates, screws, and intramedullary nails. Specific equipment included titanium alloy screws and plates (Synthes, West Chester, PA, USA) and reamed intramedullary nails (Smith & Nephew, Memphis, TN, USA). Procedures were performed under fluoroscopic guidance using a Siemens Arcadis Orbic 3D C-arm (Siemens Healthineers, Erlangen, Germany) to ensure accurate alignment and fixation.

External Fixation: This technique was applied in cases with extensive soft tissue damage using the Ilizarov technique or mono-lateral fixators (Orthofix, Lewisville, TX, USA). External fixators were assembled using carbon fibre rods and stainless steel pins, and adjustments were made intraoperatively to optimize alignment.

Skin Grafting: Split-thickness skin grafts were harvested using a Zimmer dermatome (Zimmer Biomet, Warsaw, IN, USA) set to a depth of 0.012 inches. Grafts were meshed at a 1:1.5 ratio using a Zimmer mesh graft device and secured using fibrin sealant (Tisseel, Baxter International, Deerfield, IL, USA).

Local Flap Coverage: This intervention involved the use of pedicled fasciocutaneous or muscle flaps, such as the gastrocnemius or soleus flap for proximal tibial coverage. Flap viability was assessed intraoperatively using handheld Doppler ultrasound (Hadeco Smartdop 45, Kawasaki, Japan).

Free Tissue Transfer: Microsurgical techniques were employed for free flap transfers, utilizing an operating microscope (Zeiss OPMI Pentero, Oberkochen, Germany) for the anastomosis of vessels. Flaps included the anterolateral thigh flap and latissimus dorsi flap, harvested and transferred using microsurgical instruments (Aesculap, Tuttlingen, Germany).

Considering these data, the underlying algorithm and principles dealing with these types of injuries were aimed to be determined.

In total, 29 patients underwent free tissue transfer, 37 had local fasciocutaneous and muscle flap operations, and 27 had reconstruction via split-thickness skin graft and vacuum-assisted closure. In free tissue transfer operations, the recipient’s vessel was anastomosed end-to-end to anterior or posterior tibial arteries and end-to-side to the popliteal artery. Reconstruction with latissimus dorsi free muscle flap operation was performed in 12 patients, osteocutaneous fibula free flap in 8 patients, rectus abdominis free muscle flap in 3 patients, radial forearm free flap in 3 patients, and anterolateral thigh free flap in 3 patients. Total flap necrosis occurred in three patients and partial flap necrosis in four patients. The reconstruction of these limbs was achieved via vacuum-assisted closure and skin grafting. Wound site infection was observed in 12 patients, and regression occurred following proper intravenous antibiotic treatment.

*Total count of operations:* Each patient’s total number of repetitive operations was documented. Patients were divided into those with more than three operations and those with three or fewer operations.

*Motor or sensational nerve dysfunction:* Tibial and peroneal nerve patency were examined at initial admission. An electromyography (EMG) study was performed, and those with motor nerve dysfunction were detected when the extremity was suitable.

*Lower Extremity Functional Scale at last follow-up:* After successful soft tissue coverage and bone union, functional scores were assessed by LEFS [11,12,13]. The patients were categorized into five groups of disability based on their percentage of maximal function (LEFS/80 × 100): bedbound—0% to 20% score; extreme disability—20% to 40% score; severe disability—40% to 60% score; moderate disability—60% to 80% score; minimal disability—80% to 100% score.

### 2.2. Preparation of Data

Data were extracted from electronic health records and entered into a secure database (REDCap, Vanderbilt University, Nashville, TN, USA). Data cleaning involved verifying patient identifiers, cross-referencing surgical details with operative reports, and ensuring the completeness of LEFS scores at all time points. Missing data were addressed using multiple imputation techniques, ensuring the robustness of the dataset.

### 2.3. Data Analysis

Statistical analyses were performed using SPSS version 26.0 (IBM Corp., Armonk, NY, USA). Descriptive statistics summarized demographic and clinical characteristics. The correlation between LEFS scores and variables such as MESS, Gustilo–Anderson classifications, and nerve injuries was assessed using Pearson’s correlation coefficient. Multivariate regression models were constructed to identify predictors of functional outcomes, adjusting for potential confounders such as age, gender, and number of surgeries. Statistical significance was set at *p* < 0.05.

Kaplan–Meier survival analysis was employed to estimate time to union and flap survival, with log-rank tests comparing different surgical techniques. The reliability of LEFS as a functional measure was evaluated using Cronbach’s alpha, ensuring internal consistency.

**Figure 1:**
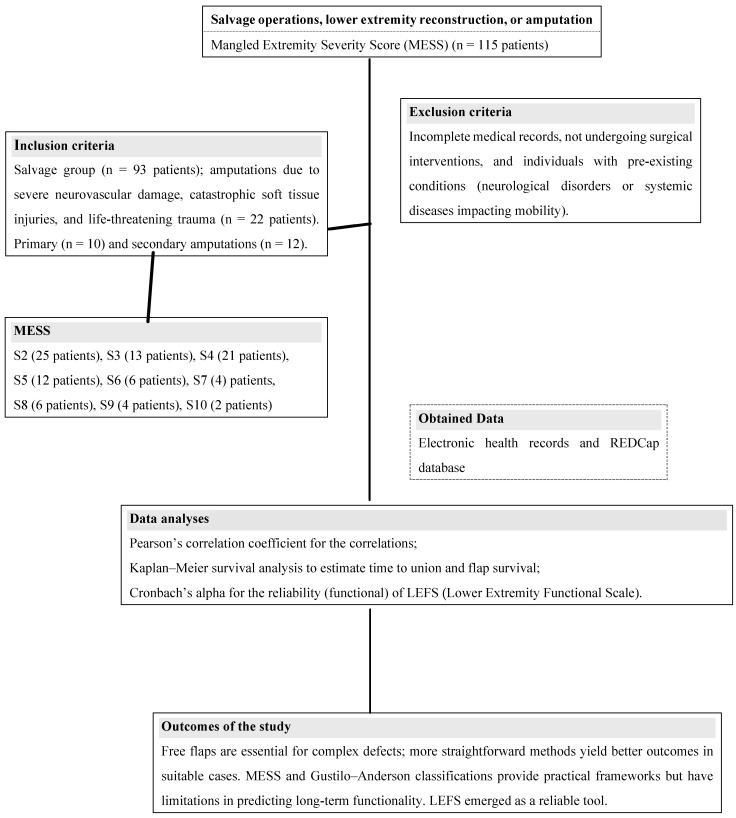
Study flowchart (S: subcategory).

This comprehensive methodology provides a detailed framework for replicating the study and facilitates future research to optimize outcomes in mangled lower extremity reconstruction. The summary of patient data is presented in Table 1, and flowchart of the study is demonstrated as Figure 1.

## 3. Results

This study collected retrospective data. The mean follow-up period was 22 months (8 to 35 months).

*Healing:* The average healing time of soft tissues was about 18 days, and the average bone union time was 17 weeks.

*A—Age:* In total, 11 patients were over 50, and 82 patients are below 50.
*B—Location of injury:* For 20 patients (22%), the injuries were in the proximal one-third, including the knee, for 28 patients (30%) in the middle one-third, and for 45 patients (48%) in the distal one-third, including the ankle. Eleven patients of the proximal and distal one-third group had an intra-articular fracture of the knee (two cases) and ankle (nine instances) joint.
*C—Mangled Extremity Severity Score:* The MESS results were 7 or above (≥7) in 16 patients and less than 7 (<7) in 77 patients.
*D—Gustilo–Anderson classification:* In total, 24 patients had grade-one fractures, 30 had grade-two fractures, and 39 had grade-three fractures.
*E—Soft tissue reconstructive and bone fixation methods performed:* Soft tissue coverage protocols were divided by assessing local or distant flap requirements. The operative procedure was executed in the first 72 h when free tissue was transferred and planned early after emergency service admission.

In case of flap failure initially, skin grafting combined with vacuum-assisted closure is preferred [5]. Local flap options and skin grafting were selected primarily in elderly patients with comorbid diseases. When the condition of recipient vascular structures is burdened by already existing vascular pathologies such as arteriosclerosis, free tissue transfer is not performed.

For defects of the distal one-third of the lower extremity, free tissue transfer procedures were performed. For proximal and middle-third defects, gastrocnemius and soleus muscle flaps, transposition flaps, and bipedicle fasciocutaneous flaps were preferred. Three patients with bone defects in the middle third underwent reconstruction with ipsilateral fibula osteocutaneous flaps.

*F—Total count of operations:* In total, 69 patients had three or fewer surgeries, 24 had more than three surgeries. High-energy open tibia fractures required an average of 8.5 months to heal.
*G—Motor or sensorineural dysfunction:* Six patients had motor nerve injuries, four in the tibial and two in the peroneal nerve. Their overall scores were poor.

Finally, the correlation of LEFS with bone healing time and free flap requirements was mainly insignificant. However, the correlation of LEFS with MESS, permanent motor nerve dysfunction, Gustilo–Andersen fracture types, and intra-articular fractures is worth mentioning.

Influence of Smoking and Surgical Complexity

Smoking was identified as a significant factor associated with delayed union and increased infection rates. Among the smokers in the study, complications were notably higher, leading to prolonged recovery periods. Additionally, patients who underwent more than three surgical interventions experienced poorer functional outcomes, as reflected in their LEFS scores (*p* < 0.05). Flap failures were also correlated with decreased functional recovery, underscoring the importance of successful surgical interventions in optimizing outcomes.

## 4. Discussion

Open fractures of the tibia are the most common open long bone fractures, with an annual incidence of 3.4 per 100,000 [14,15]. The mean age of those who sustain open tibial fractures is 43.3 years, most frequently occurring in young adult males and elderly females [16]. High-energy trauma is the primary mechanism of injury, with over 50% of cases being attributed to road traffic accidents or falls from a great height [14,15,16,17]. Noteworthy, most proximal and distal tibial fractures present with a significant soft-tissue injury and pose additional complexity when managing the injury. Primary amputation may be considered in cases of uncontrollable hemorrhage, prolonged crush injury, an avascular limb, or segmental bone/muscle loss. The decision is not taken lightly and should be discussed with other experienced surgeons [18,19,20]. Severity scores may provide a measured value in predicting the need for amputation [21,22].

Secondary amputation may also be a viable surgical option in an injury complicated by ongoing problems, e.g., deep infection. Amputation indicates poor outcomes [18], but it may be necessary to prevent further deterioration or maintain the quality of life. Long-term functional results may not be significantly affected in the amputee compared to those that would have occurred in a salvaged injury.

Open fracture management requires a careful assessment of the bone injury, including fracture characteristics and bone loss, as well as soft tissues, including contamination, integument injury, muscle damage, and neurovascular injury. The Gustilo–Anderson classification and MESS remain the most utilized classification systems for open fractures.

Managing severely traumatized limbs is challenging for orthopedic and plastic surgeons. The main aim here is not to salvage the limb but to give the patient a sensate, functional limb. Existing scoring methods to help decide whether to amputate or reconstruct the limb are considered inadequate [2]. The current literature focuses on surgical salvage of the limb rather than outcomes. Patients are deliberately dissatisfied when this demanding treatment ends with poorly functioning extremities. Is there a predictable value of salvaged limb that is functionally stable in daily life and work?

Over the past three decades, lower extremity amputation rates have gradually declined thanks to the development of microsurgical techniques and devices. Functional reconstruction of the lower extremities is now possible [3]. The goals of open fracture management include decreasing infection risk and promoting fracture union.

Fractures of bony structures and injuries to neural and vascular structures often complicate severe lower extremity injuries [3]. The vascular injuries that threaten the viability of the limb should be treated beforehand [2]. Depending on the nature of the defect, the most appropriate reconstruction procedure was chosen, and successful reconstruction results were achieved. Simple local flaps and skin grafts were the treatment modalities for children. Additional donor site morbidity may lead to functional impairment in future years. While vacuum-assisted closure and skin grafting are the reconstruction methods of choice for fundamental tissue defects, free tissue transfer is the gold standard for reconstructing composite tissue defects [3,4,23,24].

Gustilo–Anderson Grade 3C fractures and some Grade 2B fractures in elderly patients are challenging to manage [10,25]. Problems with soft tissue coverage are directly related to bone union. Good periosteal coverage is a prerequisite for bone healing. Delayed healing and non-healing are correlated strongly with soft tissue problems.

The management of mangled lower extremity injuries, characterized by complex fractures, extensive soft tissue damage, and compromised vascular integrity, remains a formidable challenge in trauma surgery. The decision-making process in such cases often hinges on the severity of the injury, as quantified by scoring systems like the Mangled Extremity Severity Score (MESS) and the Gustilo–Anderson classification. These tools aid clinicians in determining the feasibility of limb salvage versus amputation, aiming to optimize functional outcomes. Recent advances in reconstructive techniques, including local flaps, free tissue transfers, and vacuum-assisted closure, have expanded the armamentarium available to surgeons. Yet, the variability in patient outcomes underscores the need for a deeper understanding of factors influencing recovery. This study contributes to the field by examining the interplay between injury severity, surgical interventions, and functional recovery, offering insights that could inform clinical practice and future research.

MESS has been a shining star in evaluating mangled extremities [26]. But in the past decade, its deficits have become prominent, and its reliability has been damaged. The positive and negative value of prediction has also been emphasized [27].

Ankle and knee motion is critical to our series. Achieving a stable and pain-free joint in tibial fractures is paramount to patient satisfaction [28]. Therefore, every attempt should be made to restore ankle and knee joint congruency [29].

Motor nerve status is an essential aspect of the function, but the loss of sensibility of the plantar area also significantly impacts functions [30]. Thus, every attempt was made to preserve the integrity of the tibial nerve rather than the peroneal nerve. The functional outcomes of patients who underwent nerve repair were significantly worse than those who did not. In addition, it was observed that the functional scores of patients with three or more surgeries were negatively affected.

LEFS is a reliable and patient-centred assessment tool that reflects patients’ conditions from their perspective [7,31]. There is no need for any external intervention or the usage of medical devices. Patients are highly oriented and satisfied with the final scale of LEFS. Recent functional studies between amputee and salvaged patients have shown that amputee patients have higher functional scores, lower pain scores, and earlier return to daily life and work [32]. LEFS scores are strongly correlated with MESS over 7, Gustilo grade 3C fracture, free tissue transfer, fractures involving the ankle, and tibial nerve injuries. The functional scores are strongly related to MESS and Gustilo–Anderson types. These results suggest that free flaps used in delayed union groups and other groups had lower LEFS scores, while local flaps and regular union groups generally had good LEFS scores [33]. Three patients underwent repair for osteomyelitis of the tibia and developing non-union with an ipsilateral fibular bone flap. When the final values of these patients were examined, they were found to be terrible overall.

All type 3C patients underwent revascularization. In four patients, the injury was repaired at the level of the posterior tibial artery. This was because these patients also had nerve injuries in addition to vascular injuries. In four patients, vascular repair was successful, and the extremity was saved. Neural repair was unsuccessful in one and there was partial damage in the others. While six patients underwent immediate restoration, one underwent secondary repair with a sural nerve graft. The final score of patients who underwent nerve repair was less than 7.

Free tissue transfer was performed in all patients with type 3C and type 3B. The wait time for these patients was, on average, eight days longer than in the remaining cases. No significant change in final scores was observed.

So, we can create a “prediction scale” for whether salvaged limbs will be functional or non-functional [34]. Then, we can inform the patient during the course of the treatment if our efforts are likely to result in poor function even compared with amputee patients.

One significant limitation of our study is its retrospective design, which limits our ability to control for all potential confounding factors. However, this study contributes to the existing literature by encouraging future research into prospective studies in this field. It is important to note that the current literature mainly focuses on the roles of multidisciplinary teams in preventing amputations and providing post-amputation care, rather than specifically addressing the determination of amputation levels. So, comprehensive and further studies are needed for the clinical assessments.

## 5. Conclusions

The reconstruction of extensive lower extremity injuries requires the utmost theoretical knowledge and technical skill in plastic and orthopedic surgery. Successful reconstructions in these cases require the choice of the most appropriate treatment modality for the patient and the flawless execution of the procedure.

Functional score, complications of bony union, and healing time are closely related to the adequacy of treatment to cover the soft tissue. Shorter healing time and higher functional scores are better in patients with less than 7 in MESS and a lower Gustilo–Anderson classification. 

Future research should consider conducting prospective cohort studies or randomized controlled trials to validate our findings, with a particular emphasis on the long-term benefits of following multidisciplinary care (MDC) recommendations across diverse populations. Additionally, exploring the specific barriers to adherence, taking into account both patient and provider perspectives, could yield valuable insights for improving compliance rates and enhancing patient outcomes.

Future research should focus on prospective studies to validate these findings and explore the biological mechanisms underlying the observed differences in healing and function. Investigations into innovative surgical techniques and rehabilitation protocols are warranted to enhance outcomes further. Additionally, exploring the role of advanced imaging and biomarker analysis could provide deeper insights into patient-specific risk factors and recovery trajectories, ultimately leading to more refined and effective treatment paradigms. Our research evaluates the results obtained through the Lower Extremity Functional Scale (LEFS) and investigates its relationship with MESS, the Gustilo–Anderson classification, and the anatomical specifics of injuries in 93 patients.

## Figures and Tables

**Table 1:** Summary of patient data on mangled lower extremities.

Category	Subcategory	Number of Patients
Mangled Extremity Severity Score (MESS)	2	25
	3	13
	4	21
	5	12
	6	6
	7	4
	8	6
	9	4
	10	2
Gustilo Classification	I	24
	II	30
	III A	16
	III B	19
	III C	4
Soft Tissue Reconstruction	Skin graft and/or VAC	27
	Local flaps	37
	Free flaps	29
Orthopedic Procedures	Cast	17
	Intramedullary nail and plate	38
	External fixators	16
	Both	22
Functional Scores	Bedbound	0
	Extreme disability	2
	Severe disability	7
	Moderate disability	17
	Minimal disability	66

VAC: vacuum-assisted closure.

## Data Availability

The original contributions presented in this study are included in the article. Further inquiries can be directed to the corresponding authors.

## Abbreviations

The following abbreviations are used in this manuscript:

MESS	Mangled Extremity Severity Score
LEFS	Lower Extremity Functional Scale
EMG	Electromyography
